# Integral patient care: mental health in a critical patient service

**DOI:** 10.1186/cc10195

**Published:** 2011-06-22

**Authors:** LR Guastelli, ALM Silva, A Mafuf Neto, CMP Araújo, RP Blaya, ALL Camargo, E Silva

**Affiliations:** 1 Hospital Israelita Albert Einstein, São Paulo - SP, Brazil

## Objective

To determine the impact of the introduction of multidisciplinary meetings on mental health in the identification of psychiatric and psychological risks in a unit of critical patients.

## Introduction

Mental healthcare in hospital wards for critical patients is necessary both for individuals with psychological or psychiatric disorders that require intensive medical care and for those individuals who develop these disorders during hospitalization, often in the same function, illness or treatment. These disorders may cause negative impact on adherence to clinical care, well-being, psychosocial rehabilitation and patient safety during hospitalization. In our department there is a psychologist working in conjunction as part of the healthcare team, aiming to identify psychological risk factors that may impact on treatment and help the team in handling difficult situations psychologically. To identify patients with psychiatric risk, we developed a protocol for Psychiatric Risk Assessment, whereby the presence of 11 items identified by the nurse initiates the discussion of a case with a psychiatrist at the Center for Psychosomatic Medicine of Hospital Israelita Albert Einstein, which directs care and/or suggests mental health interventions. Driving this protocol is the need to ask the nurse to discuss with the mental health professional based on the identification and recovery of behavioral changes that may be missed and/or be identified only when there is already an exacerbation of psychiatric conditions or occurrences related to them. Aiming to assist the nursing staff on early identification of these risks and organize actions during the stay in the ward and at discharge, a multidisciplinary meeting weekly was implemented to discuss cases and situations related to them.

## Methods

Implementation of a multidisciplinary meeting consisting of nurses, psychologists, psychiatrists, medical and nursing coordinators in November 2010. Conducting a weekly meeting with the purpose of discussing situations related to behavioral changes in patients hospitalized in the unit, planning, multidisciplinary care and management of cases. After the meeting, the nurse forwarded to the treatment team a summary that included a description of what qualifies as a psychological or psychiatric risk factor for each case, the guidelines for the team for management of the situation and suggestions for the doctor when involving medical management.

## Results

There were 68 psychiatric risks in the semi-intensive unit in the second half of 2010. Of these, 31 cases were reported in December, the month following the beginning of the multidisciplinary meeting. Whereas 12 cases were reported in October and 12 cases in November, there was an increase of 158% in the number of cases reported in December. Regarding reports of psychological risk, we observed that the multidisciplinary meeting to discuss the risks promotes to the nurse the understanding of all aspects involved, allowing the discrimination of the psychological aspects and relevance to specialist interventions as well as instrumentalizing the team to handle the patient and family.

## Discussion

The discussion of disciplinary cases seems to have enabled an understanding, appreciation and discrimination of which behaviors observed by the nurse should be accompanied by the psychology team as the protocol of psychiatric risk. The discussion of mental health with professionals may have afforded the team a better idea of how these professionals can help provide routine care, promoting the early identification of psychological and psychiatric risks. Other studies should be performed to confirm the effectiveness of this intervention.

## Conclusion

A multidisciplinary meeting was effective to assist the team in early detection and recovery of his observations of psychiatric disorders in hospitalized patients in a semi-intensive unit.

